# Management of Hemoglobin Disorders During the COVID-19 Pandemic

**DOI:** 10.3389/fmed.2020.00306

**Published:** 2020-06-09

**Authors:** Sanjana Fatema Chowdhury, Saeed Anwar

**Affiliations:** ^1^Department of Genetic Engineering and Biotechnology, Shahjalal University of Science and Technology, Sylhet, Bangladesh; ^2^Department of Medical Genetics, Faculty of Medicine and Dentistry, University of Alberta, Edmonton, AB, Canada

**Keywords:** hemoglobinopathies, sickle cell diseases, thalassemia, hemoglobin, iron chelation therapy, COVID-19, SARS-CoV-2, clinical management

## Abstract

The coronavirus disease 2019 (COVID-19) is an emerging infectious disease that has become a global public health concern after being first reported in China and has subsequently spread worldwide. It causes mild to severe respiratory illness with some flu-like symptoms. The causal virus behind this disease, SARS-CoV-2 (severe acute respiratory syndrome coronavirus 2), conceivably attacks the receptors of the respiratory system of the human body but has no strict evidence of attacking the blood cells yet. However, patients with hemoglobin disorders (e.g., sickle cell anemia, thalassemia) are vulnerable to this global health situation due to their clinical complications. Such patients are generally more prone to viral and bacterial infections, which can worsen their physical condition. Some of these patients present immunocompromised conditions, e.g., splenectomized or post-transplant patients. Therefore, they should follow some preventive steps such as shielding as well as the general guidelines for the COVID-19 pandemic. Transfusion dependent patients require regular monitoring for iron overload, and iron chelation therapy may be stopped by the physician depending on the situation. This article reviews the management strategies and provides some crucial recommendations for people in the corner with hemoglobin disorders.

## Introduction

The outbreak of coronavirus disease 2019 (COVID*-*19) began in Wuhan, China, in late 2019 and continues to spread globally. In around 6 months after the emergence of severe acute respiratory syndrome coronavirus 2 (SARS-CoV-2), the causal agent of COVID-19, over 4.5 million positive cases, and 310 thousand deaths have been reported. Risk factors for adverse outcomes include advanced age, comorbidities (e.g., obesity, diabetes, cardiovascular, pulmonological and renal diseases), and male sex (1, 2). Also, people with weakened immune systems from a medical condition or treatment are at a higher risk. Clinicians, researchers, academicians around the globe, are working hard to find an effective vaccine or treatment strategy against the disease. However, there is still uncertainty of management and mitigation strategies for the patients who need critical care and effective treatment. Researchers and clinicians have so far recorded only a dearth of reports of infected patients with hemoglobin disorders. Because of the limited clinical evidence, any statement on these subjects may be regarded as a mere theory but cannot be ignored. However, as the virus is rapidly spreading, cautionary thoughts about the factors which may render these patients fragile in front of this infection are necessary.

In this review, we briefly highlighted why individuals with hemoglobin disorders belong to vulnerable groups for COVID-19 infection. It also provides some recommendations on how the physicians and health care professionals could continue the management interventions for individuals with hemoglobinopathies during the COVID-19 pandemic.

## Sickle Cell Disease

Individuals with sickle cell disease (SCD) are a vulnerable group of patients, with a higher risk of severe complications than the general population (3). Compared to the general population, individuals with SCD have a relatively higher risk for acute pulmonary illness as well as viral infections (4). Hospitalization records of two influenza seasons (2003–2005) showed that children with SCD were 56 times more frequently hospitalized than children who did not have SCD in four states of the United States (5). Surprisingly, the rates were twice higher than those of children with cystic fibrosis, a genetic disease characterized by the buildup of thick, sticky mucus, which causes severe damage to the respiratory and digestive systems (5). An elevated need for intensive medical care was also observed in these patients. Moreover, any respiratory infection-related hypoxia, dehydration, or acidosis may provoke a vaso-occlusive crisis as well as acute chest syndrome (ACS). Existing evidence suggests that ACS is a major cause of death in individuals with this hereditary blood disorders (6).

As patients with SCD usually have a higher risk of respiratory complications, and SARS-CoV-2 potentially causes severe respiratory complications, it may cause even more severe complications in SCD patients (7). Furthermore, a common drug that people with SCD are usually administered is hydroxycarbamide (hydroxyurea), which possibly have an immune-compromising effect (8). Although there is yet any evidence on the prevalence and severity of known hydroxyurea-associated viral infections in individuals with SCD, it cannot be ignored entirely as a causal factor to put these people at a disadvantage.

## Thalassemia

Thalassemia is another hemoglobin disorder in which the body produces an abnormal or inadequate amount of hemoglobin. Unlike in SCD, individuals with thalassemic conditions do not possess an increased risk of lung infections (9). Some other comorbidities, including heart disease, liver disease, diabetes, and severe iron overload, may make them vulnerable to the virus, especially in adults. Transfusion-related excess iron load in blood plasma results in non-transferrin bound iron to enter some cells and convert to ferritin, which over time, becomes hemosiderin. If the hypothalamus or adrenal glands are affected with excess iron, it can result in adrenal hypofunction (10) which may limit the effects of infections or the ability to fight infections may be compromised. A recent study conducted in Italy analyzed a cohort of 11 individuals with thalassemic conditions did not observe increased severity of COVID-19, which is, perhaps, due to quicker and vigilant self-isolation compared to the general population (11). However, such a preliminary study does not imply that individuals with thalassemia may not develop severe forms of COVID-19. A larger cohort of patients may define the impact of the disease in individuals with thalassemic conditions. Until then, physicians, health-care professionals, and caregivers should give special consideration when dealing with COVID-19 patients with thalassemia. The potential of low-dose vitamin supplementation to help treat the anemic condition in these patients may also be evaluated. Moreover, it must not be ignored that complications may be increased due to the slowdown of the clearance of SARS-CoV viral RNA from the respiratory tract by corticosteroids (12, 13).

A recent study held in Italy showed that the prevalence of heterozygous ß-thalassemic condition correlates to immunity against COVID-19 (14). Using a multiple linear regression analysis, it proposes that individuals with heterozygous ß-thalassemic conditions may develop immunity to SARS-CoV-2 viral infection. However, this study relied solely on statistical methods, and the study design had several limitations (e.g., study period, the presence of ß-thalassemia heterozygotes, and other co-factors in the sample). Although there is yet any strong clinical evidence and validating studies to support this hypothesis, patients with thalassemic conditions should be taken care of with extra caution.

## Impacts of Covid-19 in Hemoglobin

Hemoglobin is a red blood cell protein comprising four globular protein subunits carried by an embedded heme group. An iron ion and another porphyrin compound comprise a heme group. Red blood cells deliver oxygen in the lungs and deliver it to the rest of the body through the reversible binding of oxygen to the iron ion in heme groups.

A recent study on blood indices in individuals with COVID-19 infection revealed that most patients had a normal complete blood count and Lactate Dehydrogenase (LDH) on admission. None of the patients presented moderate or severe thrombocytopenia, which is a common finding in other viral illnesses, e.g., dengue fever (15). Though a meta-analysis on observed heterogeneous available studies showed that in severe diseases of COVID-19 compared to those with milder forms, hemoglobin values are substantially reduced, thus confirming previous evidence garnered from patients with other types of pneumonia (16).

Computational proteomic analysis revealed that some SARS-CoV-2 proteins could bind to porphyrins. In contrast, three other viral proteins attack the heme on the 1-ß chain of hemoglobin, resulting in the dissociation of iron to form porphyrin (17). Deoxyhemoglobin is at a higher risk of viral attacks than oxidized hemoglobin, which may cause respiratory distress symptoms because of lesser oxygen carrier hemoglobin in the body. However, there is no experimental evidence to date to support the conclusions of this computational analysis. Outcomes of computational studies often cannot hold up when interpreted according to acceptable standards, yet represent points of concern (18). Wet-lab studies to validate the hypothesis could be a timely topic of interest for many researchers.

Any significant decline in the hemoglobin concentration, whether gradual or drastic, may result in a worse clinical outcome. Hence, preliminary evaluation and monitoring of hemoglobin values in patients with the SARS-CoV-2 infection should be considered. The physicians and the front-line health-care professionals caring for patients with thalassemia should consider whether transfusion support, e.g., administration of blood or packed red blood cells would improve the condition of clinically complex conditions. The interaction between hemoglobin and SARS-CoV-2 would be a focus of many future research endeavors.

## Transfusion Transmitted Risks of SARS-CoV-2

Coronaviruses primarily cause mild to severe respiratory infections, and there is yet any evidence of transfusion-related transmission from either SARS-CoV or MERS-CoV and SARS-CoV2 viruses (19, 20). Nonetheless, the possibility of transfusion-related transmission of COVID-19 cannot be ruled out either. Theoretically, a blood donor with asymptomatic infection who has not been tested may contribute to the spread of the virus unknowingly. If a person gets in contact with a confirmed patient or anyone who referred to traveling from COVID-19 affected area, they should be deferred for the donation of blood for 21 days following exposure (same applied for SARS-Cov and MERS-CoV). COVID-19 patients who have confirmed recovery should be deferred from blood donation for at least 28 days after being asymptomatic and completion of medication (21). Some precautionary measures should be followed during the current outbreak, such as taking body temperature before blood donation, asking the donors other physical symptoms and travel history within 28 days, and keeping the donors on a check after donation (22).

In some cases, viral RNA and virus-related antibody screening of blood donations or the use of pathogen inactivation/reduction technologies (PRT) may be conducted. However, no single PRT is suitable for all blood products because some blood components may damage due to PRT treatment (23). Moreover, extracorporeal membrane oxygenation (ECMO) services can be considered for the severe acute respiratory distress syndrome (ARDS) patients in critical care during this pandemic (24). In an observational study, a higher survival rate is reported for acute respiratory distress syndrome patients in an ECMO settings (24).

Patients, caregivers, and the donor should maintain a certain safety distance to prevent transmission. Nevertheless, as a large group of people with hemoglobin disorder are dependent on regular blood transfusion (25), it has been a matter of concern. The volume of blood donation has declined since the emergence of COVID 19 (26). Outdoor blood donation programs are not happening now (27). Also, many people, who are practicing Muslims, including the regular and registered donors, avoid donating blood during Ramadan and immediately after Ramadan. The extended and unplanned lockdowns are, however, affecting the movement of blood donors and patients from rural areas of the country. So, there is a double challenge for the patients who cannot avoid blood transfusion.

Blood banks should take initiatives to engage more volunteer donors after proper screening. Country-specific pro-active measures are to be taken, as the COVID-19 situation seems to sustain for longer (28). As hospitals, clinics, and health care facility premises are highly infectious environments, hospitals should consider isolating patients with hemoglobin disorders from suspect COVID-19 cases and creating a separate triage area for those living with these conditions. Simple (top-up) transfusions can be used in some patients, as they involve significantly fewer blood units than exchange transfusion. Simple transfusion can be another alternative for patients with hemoglobin disorders because it involves a lot of fewer blood units than exchange transfusion (28). In order to keep the vulnerable transfusion-dependent patients within a safety net, community-based blood banking needs to be encouraged.

## Management of Iron Chelation Therapy

It is recommended for all transfusion-dependent thalassemia major patients to stop iron chelation therapy when the patient feels febrile until the fever has resolved, and the cause of fever has been medically assessed. However, under some circumstances, particularly when cardiac iron is increased (29), stopping chelation in fever can be harmful. So, each case must be reviewed by a clinician familiar with thalassemia and chelation management.

Patients self-isolating due to symptoms or an infected household member does not know if they have COVID-19 or not. They will need to contact their transfusion doctors and consultants to plan or postpone transfusion therapy according to their isolation period.

Iron overload and effects of iron chelation monitoring should be continued regularly. Patients who need regular transfusions should follow an outpatient review at the same time as transfusions. It should be considered if routine MRI monitoring for iron overload can be delayed in stable patients. In the feverish condition, chelation agents should be stopped at once, and the physician is in regular contact for advice.

## Management of Patients Having a Stem Cell Transplantation or Gene Therapy

Hematopoietic stem cell transplantation (HSCT) and gene therapy is the treatment of choice for patients with hemoglobin disorders. They require more intensive and prolonged post-transplant immunosuppression because infections caused by endogenous herpesviruses, human herpesvirus (HHV)-6, BK virus, and respiratory viruses are increasingly reported (30). It is still unclear whether immunosuppression related to transplantation changes the odds of acquiring infection with SARS-CoV-2 or if the disease implications are altered. It cannot be denied that the clinical expression of disease could be diminished by the anti-inflammatory effects of immunosuppression as well (31). Until structured studies are reported to enhance the understanding of this disease pathway and process, some precautionary measures should be taken for this type of patient.

The transplant clinicians should give major concerns to the patients in need of an urgent transplant, including testing of donors, decisions on organ suitability from recently exposed or infected donors, and the implications of recovery of such organs. At this time, it would be wise to avoid transplantation of organs from donors with contact to an affected patient or travel history to an area with a high density of infection. For non-urgent, non-malignant conditions (SCD and thalassemia) transplantation and gene therapy should be stopped until the situation stabilizes. Diagnostic checkup investigations should be put on hold for pre-transplantation. Sickle cell disease or thalassemia patients who have had hematopoietic stem cell transplant or gene therapy or any patient having a stem cell transplant and is still on immunosuppression should strictly shield regardless of the timeline passed by since the procedure.

## Management of Patients in Marginalized Communities

Only 5% of the population having hemoglobin disorders live in countries with optimal care, and the remaining 95% are either treated suboptimally or remain outside of any treatment coverage (32). Concerns grow about the South Asian and some Mediterranean countries, who are part of the worlds so called “thalassemia belt” (33), as they have large populations with hemoglobin disorders and, often, inadequate health infrastructure. The government of such regions should encourage community blood banking among healthy individuals to maintain a sufficient blood supply for weeks to come in this interminable crisis. Transfusion dependent patients and their guardians should not only depend on blood banks but also find at least a few donors such as family or friends who could help them. The national and international groups should pay more attention to these patients in marginalized populations. Other treatment facilities, such as chelation therapy (if unavoidable), should be made available to them by the health workers or volunteers. There is no known end date in this fight against coronavirus, so extra care should be taken to prevent person to person spread of coronavirus.

## Recommendations

The COVID-19 is a rapidly evolving and emerging situation. There is a dearth of information for people with hemoglobin disorders in the context of COVID-19. Patients with hemoglobin disorders are strictly recommended to follow shielding to protect them by reducing interaction with other people (34).

The general protocols (35) are to be maintained strictly for all, including the patients with hemoglobin disorders, while some recommendations should be specifically for them. People of extremely vulnerable groups like them (sickle cell disorder or thalassemia) must always stay at home, and unless an emergency, they should avoid any direct contact. They should strictly avoid contact with someone displaying symptoms of coronavirus (COVID-19)—a fever, and the temperature above 38 degrees with symptoms such as dry cough, fatigue, dyspnea, and difficulty breathing (36). They can maintain communication through remote technologies, for example, the internet and social media.

Patients showing symptoms of cough, fever, fatigue, or other respiratory symptoms such as breathing problems or sore throat, should be tested for COVID-19 and other respiratory viral pathogens as well. Sickle cell disease and thalassemia patients having respiratory symptoms should have a chest X-ray (37). Also, sickle cell disease patients admitted for a vaso-occlusive crisis should also have chest X-rays. COVID-19 positive patients or patients with chest X-ray showing pulmonary infiltrates suggestive of ACS (Acute Chest Syndrome) in SCD, should be hospitalized for intensive care (7), and ACS guidelines should be followed to manage them. A low dose of hydroxyurea is recommended in children with sickle cell anemia who need regular blood transfusion therapy to prevent primary or secondary stroke (38). This is especially needed in areas with severe blood shortages because hydroxyurea treatment will decrease acute vaso-occlusive pain and ACS events in the absence of regular blood transfusion therapy (38). Although pediatric COVID-19 patients show a milder clinical course compared to adults, some reports show thrombosis in COVID-19 patients (39). Therefore, prophylactic doses of anticoagulant are given to all SCD patients with severe COVID-19 unless full anticoagulation symptoms are observed (39).

All individuals with hemoglobin disorders should maintain a healthy, balanced, and immune boosting diet within their guidelines, as prescribed by their attending physicians. Individuals with hemoglobin disorders may consider eating fresh and unprocessed foods, e.g., fruits, vegetables, legumes, nuts, and whole grains every day, which would provide them with vitamins, minerals, fibers and antioxidants. While eating increased amounts of vegetables and fruits is good for immunity, eating too much of them can lead to leaching of important vitamins. Also, the dietary restriction of iron and vitamin-C should be taken in consideration. Consumption of unsaturated fats, white meat, oily fish, and reduced-fat-milk is recommended. Increased amount of sugar and salts, concentrated juices and carbonated beverages, alcohols should be avoided.

Moreover, therapeutic anticoagulation strategies should also be taken into consideration after validation. Besides, if a thalassemia patient becomes COVID-19 positive, intensive care should be prepared for their admission; the treating hematologist should also be notified. The medical staff of intensive care and the patients' treating physician should maintain close communications with them (40).

## Conclusion

With the worldwide spread of COVID-19, unprecedented challenges and threats have emerged for patients with hemoglobin disorders. To date, no detailed study has been conducted on this topic, and our knowledge regarding the association between hemoglobin disorders and COVID-19 is still inadequate. Henceforth, the COVID-19 requires careful approaches for the mitigation, timely detection, and appropriate therapeutic intervention for vulnerable hemoglobinopathy patients. Moreover, profound observation of such cases and research involving the interaction between hemoglobin and the virus itself should be prioritized. Advanced epidemiological, molecular, and computational studies on the association between the SARS-CoV-2 and hemoglobin disorders, should pave the way for further analysis.

## Author Contributions

SA conceived and designed the study. SC wrote the first draft. SA and SC edited and revised the subsequent drafts. Both authors reviewed and endorsed the final submission.

## Conflict of Interest

The authors declare that the research was conducted in the absence of any commercial or financial relationships that could be construed as a potential conflict of interest.
